# Self-assembled platinum nanoparticles on sulfonic acid-grafted graphene as effective electrocatalysts for methanol oxidation in direct methanol fuel cells

**DOI:** 10.1038/srep21530

**Published:** 2016-02-15

**Authors:** Jinlin Lu, Yanhong Li, Shengli Li, San Ping Jiang

**Affiliations:** 1 School of Materials and Metallurgy, University of Science and Technology Liaoning, Anshan 114051, P.R.China; 2 Fuels and Energy Technology Institute & Department of Chemical Engineering, Curtin University, Perth, WA 6102, Australia

## Abstract

In this article, sulfonic acid-grafted reduced graphene oxide (S-rGO) were synthesized using a one-pot method under mild conditions, and used as Pt catalyst supports to prepare Pt/S-rGO electrocatalysts through a self-assembly route. The structure, morphologies and physicochemical properties of S-rGO were examined in detail by techniques such as atomic force microscope (AFM), transmission electron microscopy (TEM) and X-ray photoelectron spectroscopy (XPS). The S-rGO nanosheets show excellent solubility and stability in water and the average particle size of Pt nanoparticles supported on S-rGO is ~3.8 nm with symmetrical and uniform distribution. The electrocatalytic properties of Pt/S-rGO were investigated for methanol oxidation reaction (MOR) in direct methanol fuel cells (DMFCs). In comparison to Pt supported on high surface area Vulcan XC-72 carbon (Pt/VC) and Pt/rGO, the Pt/S-rGO electrocatalyst exhibits a much higher electrocatalytic activity, faster reaction kinetics and a better stability. The results indicate that Pt/S-rGO is a promising and effective electrocatalyst for MOR of DMFCs.

Graphene is a single-atom-thick sheet of hexagonally arrayed sp^2^-bonded carbon atoms. It is the thinnest and strongest materials ever reported. Graphene promises a great diversity of applications owing to its unique electronic, mechanical, thermal and chemical properties^1^,^2^,^3^. The earlier fundamental researches were initiated from the micromechanical cleavage of graphite for high-quality graphene sheets in a controlled and scalable manner^4^. Reduced graphene oxide (rGO) sheets can be prepared in large quantity through chemical reduction of graphene oxide (GO)^5^,^6^,^7^. However, the rGO often experiences an irreversible agglomeration and precipitation during the chemical reduction process. Extensive efforts have been made to overcome these problems. Stankovich *et al*.^8^,^9^ synthesized the stable aqueous dispersions of graphitic nanoplatelets coated by poly(sodium-4 styrene sulfonate). Reduced graphene nanosheets via non-covalent functionalization with a conducting polymer dispersant showed the decrease in the agglomeration^10^. Zhang *et al*.^11^ prepared conjugated-polyelectrolyte (CPE) functionalized rGO and the as-prepared CPE-functionalized rGO exhibited excellent solubility and stability in many polar solvents. However, the presence of the polymeric dispersing agent in graphene composite may be undesirable for some applications and alternative approaches have been explored. For example, Samulski *et al*.^12^ synthesized the lightly sulfonated graphene from graphene oxide in a multi-step process. Yang *et al*.^13^ synthesized chemically converted graphene nanosheets with 3-aminopropyltriethoxysilane, showing improved water solubility. Nano-sized graphene can also be rapidly synthesized by an ion-exchange method^14^.

Direct methanol fuel cells (DMFCs) are very promising clean power sources for portable applications owing to their relatively high energy density, low operating temperatures and ease of handling of liquid fuel^15^,^16^. Storage and transportation of liquid methanol is much easier than gaseous hydrogen as it does not need high pressure or low temperature. One of the most important reactions in DMFCs is the methanol oxidation reaction, MOR^17^,^18^. Currently, platinum-based material is still the most widely investigated and important electrocatalyst for MOR, however, it is easily poisoned by adsorbed CO, an intermediate species of the MOR and the very high costs of precious Pt-based electrocatalysts inhibit the widespread use and applications of DMFCs^19^,^20^. However, the efficiency and electrocatalytic activity of Pt-based electrocatalysts can be significantly enhanced by fine dispersion and distribution of Pt nanoparticles (NPs) and interaction between the electrocatalysts and catalyst supports^21^,^22^. The nature and choice of the support can have significant effects on the catalytic properties of the electrocatalysts^23^,^24^,^25^,^26^. Chung *et al*.^27^ prepared Pt/graphene using a pulsed galvanostatic electrodeposition method and showed the improved electrocatalytic activity for MOR as compared with a commercial Pt/C catalyst, which was attributed to the synergistic effects between the Pt NPs and graphene sheets. Shang *et al*.^28^ prepared porous graphene films (GNFs) using chemical vapor deposition and deposited Pt NPs on GNFs by magnetron sputtering. The excellent electrocatalytic activity was ascribed to the porous honeycomb-like surface structure, large surface area and enhanced interactions between graphene and Pt NPs. Pt/graphene electrocatalysts were also synthesized through a chemical reduction route using ethylene glycol as reducing agent and showed a high electrocatalytic activity for MOR^29^. Zhao *et al*.^30^ synthesized graphene and nitrogen-doped graphene (N-G) using sodium borohydride and hydrazine as reducing agents, respectively. The Pt/N-G catalyst shows a higher electrocatalytic activity for MOR owing to the smaller particle size and modulated electronic properties of the deposited Pt on N-G. Sun *et al*.^31^ synthesized boron-doped graphene (BG) through a thermal annealing method and fabricated Pt/BG catalyst via a microwave-assisted reduction in ethylene glycol solution, showing an enhanced performance of Pt/BG for MOR. It has been demonstrated extensively that graphene and GO based materials are effective electrocatalyst supports in fuel cells^32^,^33^,^34^,^35^,^36^,^37^,^38^.

Although there are many reports on the use of graphene as Pt catalyst supports for MOR, however, it is still important to further enhance the electrocatalytic performance of Pt/rGO and to explore the new applications of graphene through functionalization. In this work, we developed a facile method to synthesize sulfonic acid-grafted graphene oxide, S-rGO, as effective catalyst supports to prepare Pt/S-rGO electrocatalysts via a self-assembly route. The electrocatalytic properties of Pt/S-rGO catalysts were investigated in detail. The results indicate that the Pt/S-rGO possesses a much higher electrocatalytic activity and better stability towards MOR of DMFCs, as compared with Pt on high surface area Vulcan carbon and on rGO (Pt/VC and Pt/rGO) electrocatalysts.

## Results and Discussion

**Figure 1:**
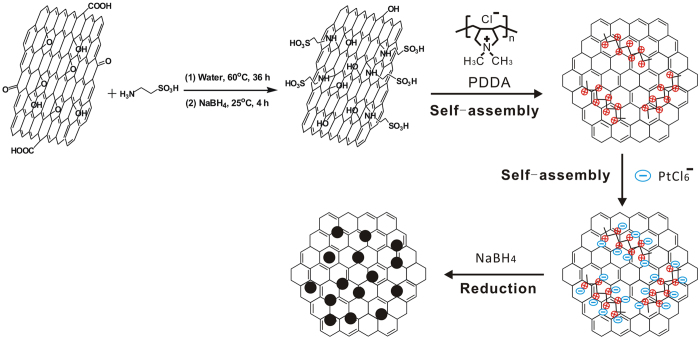
Schematic diagram of the synthesis of S-rGO and Pt/S-rGO.

It is well known that GO contains a large amount of oxygen-containing groups such as hydroxyl, epoxy, carboxyl and carbonyl groups, which open up a versatile avenue for a variety of chemical transformations^39^. Among these groups, epoxy groups on the basal planes of GO can easily react with amines through a ring-opening reaction. In this work, 2-aminoethanesulfonic acid (taurine) was used to functionalize the GO. The process was carried out in aqueous solutions without adding additional organic solvents or surfactants that may generate undesirable byproducts or residues attaching on the surfaces of GO^7^,^40^. The zeta potential (*ζ*) values of the VC, GO, rGO and S-rGO suspensions were measured and the results are listed in Table 1. The zeta potential of VC is −24.8 mV, close to −27.6 mV measured on GO. The S-rGO aqueous suspension at a concentration around 0.01 mg mL^−1^ under neutral conditions has a zeta potential of −62.4 mV, which is much lower than −10.4 mV observed on rGO, indicating the success of the fuctionalization by taurine and the very good stability of the suspension^12^,^41^. Moreover, there is no sign of coagulation of S-rGO sheets after storing in water for 3 months (Fig. S1), indicating the high dispensability of S-rGO in water. Owing to the existence of negatively charged sulfonic acid group on the surface of S-rGO, self-assembly of the added positively charged polycation, poly(diallyldimethylammonium chloride) (PDDA) occurs as the result of the electrostatic interaction^42^. Thus, the charge inversion takes place on the S-rGO with the self-assembly of the positively charged PDDA monolayer, which can function as anchoring sites for negative charges or ligands. This leads to the self-assembly of the negatively charged Pt precursors, PtCl_6_^2−^, forming an uniformly distributed Pt precursors on the surfaces of S-rGO. After reduction treatment in NaBH_4_ solution, uniformly distributed Pt NPs on the S-rGO supports were obtained. The one-pot synthesis route for S-rGO and Pt/S-rGO is shown in Fig. 1.

**Figure 2:**
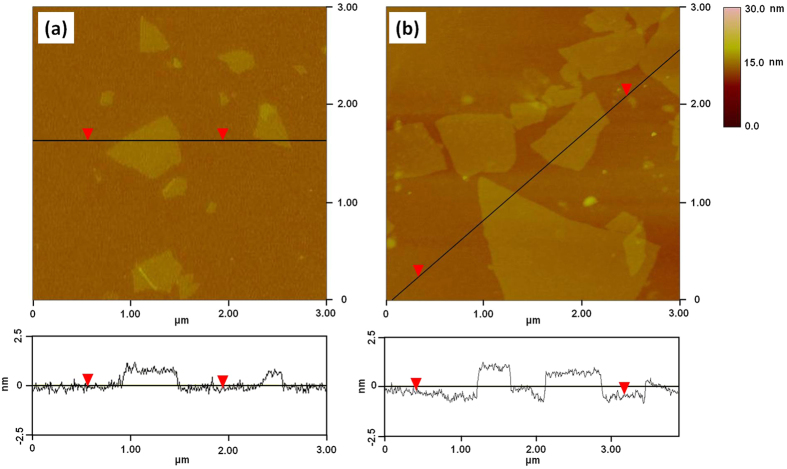
AFM images and cross-sectional analysis of (**a**) GO and (**b**) S-rGO.

**Figure 3:**
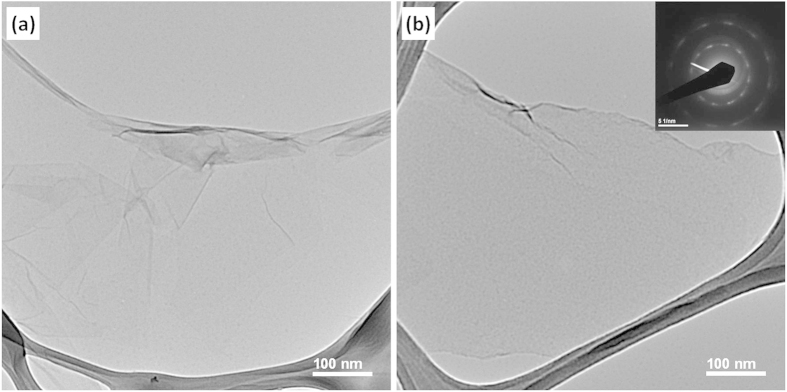
TEM images of (**a**) GO and (**b**) S-rGO. The inset picture in the top-right part of (**b**) is the SAED image of S-rGO.

The morphologies of the GO and S-rGO were examined in details by AFM and TEM. The AFM images confirm that the GO and S-rGO are comprised of isolated and well dispersed GO sheets, as shown in Fig. 2. The GO sheets have lateral dimensions of several micrometers and a thickness of 0.8 nm, which is characteristic of a fully exfoliated GO sheet^12^. The thickness of a single-layer S-rGO sheet is about 1.2 nm. This implies that the taurine groups have been grafted onto GO, giving rise to the thickness increase. The single-layer sheets can also be observed clearly for both GO and S-rGO by TEM (Fig. 3). The corresponding selected-area electron diffraction (SAED) pattern of S-rGO coincides well with the typical SAED pattern of a single layer of rGO (inset in Fig. 3b)^43^.

**Figure 4:**
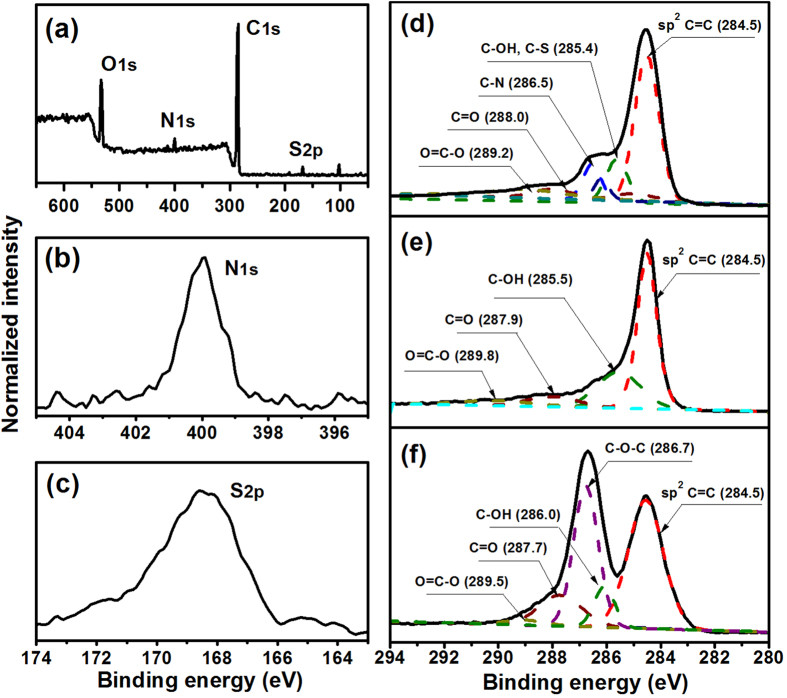
(**a**) XPS survey scan of S-rGO, XPS high resolution scan of (**b**) N1s, (**c**) S2p and (**d**) C1s of S-rGO, and XPS high resolution scan of C1s of (**e**) rGO and (**f**) GO.

Figure 4 shows the XPS spectra of GO, rGO and S-rGO. As shown in the XPS survey scan of S-rGO, the peaks related to S2p at 168.5 eV and N1s at 399.8 eV can be clearly observed in the spectra of S-rGO (Fig. 4a). The deconvolution for C peaks was carried out by using an XPSPEAK software. A Shirley type background was used and the *s* peak type was selected. Adjustable parameters for the fitting were peak position, full width at half maximum, peak area and % Lorentzian-Gaussian. Four types of carbons were identified: C-O-C (286.7 eV), C-OH (286.0 eV), C=O (287.7 eV) and O=C-O (289.5 eV) in the spectra of the GO (Fig. 4f), which are in good agreement with the previous reports^13^,^44^. After the reduction, the peak intensities from oxygen-related groups decrease significantly (Fig. 4d,e). The peaks at 286.5 eV as shown in Fig. 4d can be attributed to the C-N groups. The results indicate that taurine molecules have been successfully grafted to the GO, consistent with the AFM examinations (Fig. 2).

**Figure 5:**
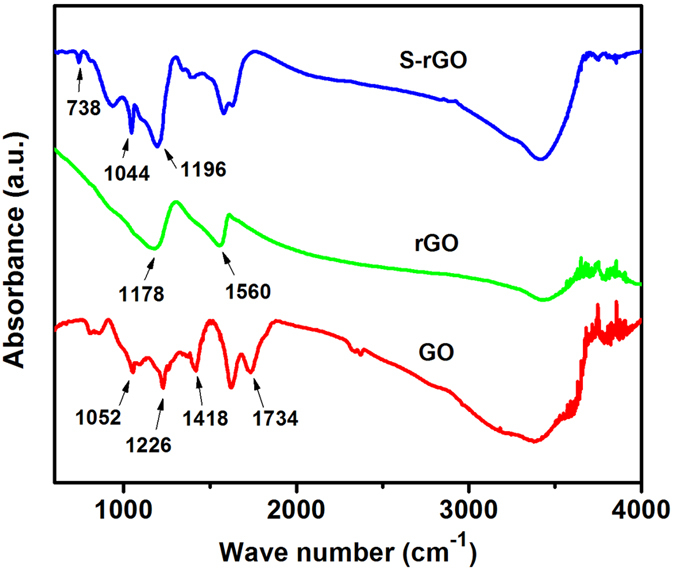
FTIR curves of GO, rGO and S-rGO.

Figure 5 shows the Fourier transform infrared spectroscopy (FTIR) spectra of GO, rGO and S-rGO. The spectrum of GO illustrates the presence of C-O (*v*_C-O_ at 1052 cm^−1^), C-O-C (*v*_C-O-C_ at 1226 cm^−1^), C-OH (*v*_C-OH_ at 1418 cm^−1^), and C=O in carboxylic acid and carbonyl moieties (*v*_C=O_ at 1734 cm^−1^). For the rGO, there are only two peaks at 1178 cm^−1^ and 1560 cm^−1^, indicating that the GO has been effectively reduced by NaBH_4_ in mild conditions. For S-rGO, the absorbance bands at 1044 cm^−1^ and 1196 cm^−1^ can be attributed to the symmetric and asymmetric stretching band of -SO_3_ groups^45^,^46^, which evidently demonstrate the successful grafting of taurine molecules on the GO and are in good accord with the AFM, XPS and zeta potential results. Raman spectroscopy for the graphitic modes of carbon provided additional evidence for the structural changes caused by the reactions (Fig. S3). The samples VC, GO, rGO and S-rGO all exhibit D band at around 1586 cm^−1^, which corresponds to a splitting of the E_2g_ stretching mode and reflects the structural intensity of the sp^2^-hbridized carbon atoms, and G band at around 1332 cm^−1^. However, S-rGO shows slightly higher D/G intensity ratio than that of rGO, implying that more small domains of sp^2^ structure were created, which is consistent with previous reports on functionalized graphene^5^,^47^.

Elemental analysis data (Table 1) indicate that the C, O and H contents in the VC, GO and rGO are within the range reported in literatures^5^,^47^,^48^. The N and S content in S-rGO is 2.3 and 5.2 wt.% of the total mass of S-rGO, respectively, and is evidently due to the grafted taurine molecules. This result indicates that taurine molecules exist in the as-prepared S-rGO composites. The electrical conductivities (*σ,* S cm^−1^) of the VC, GO, rGO and S-rGO films were measured by four-point probe method and are also listed in Table 1. The pristine GO shows a conductivity of ~2.4 × 10^−4^ S cm^−1^. The electrical conductivity of S-rGO is 4.3 S cm^−1^, exhibiting a four-order magnitude increase in comparison with that of GO, although it is slightly lower than 5.2 S cm^−1^ of rGO. The results further verify that the π-conjugated system has been effectively restored in S-rGO after the reduction^49^.

**Figure 6:**
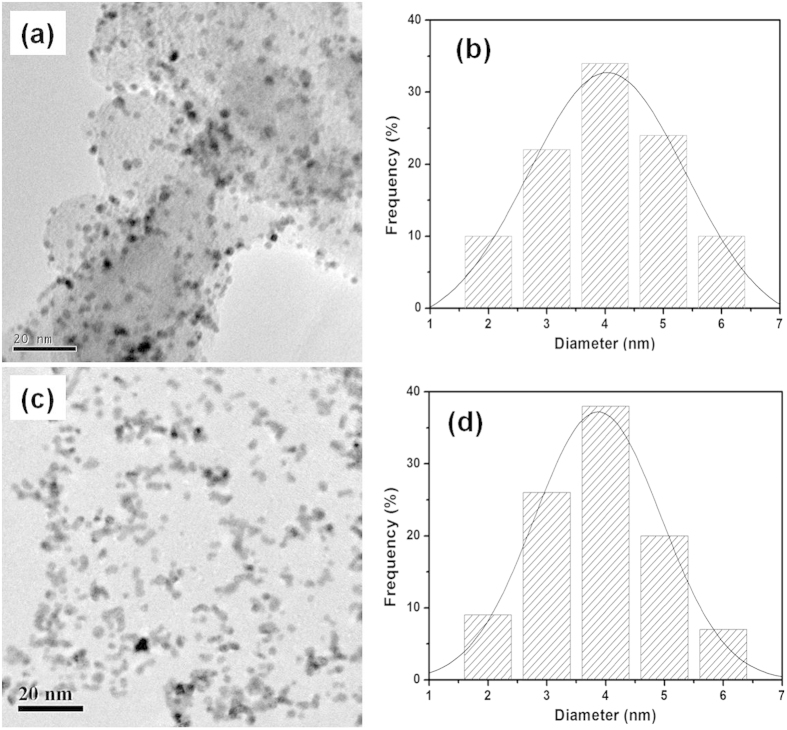
TEM images of (**a**) Pt/VC and (**c**) Pt/S-rGO, and histograms of size distribution of Pt nanoparticles of (**b**) Pt/VC and (**d**) Pt/S-rGO. Pt loading was 20 wt.%.

**Figure 7:**
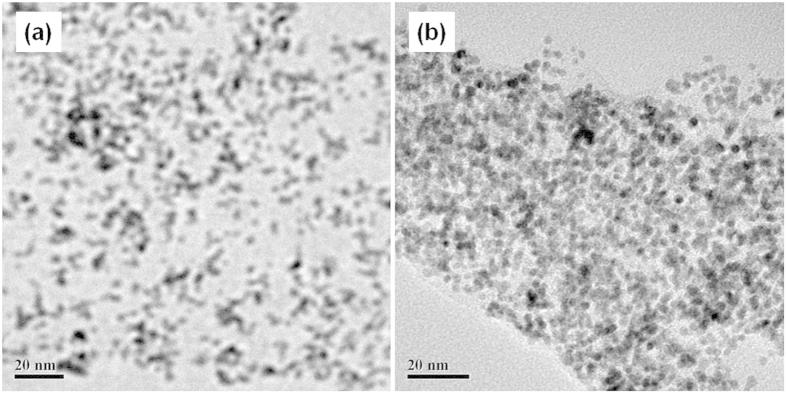
TEM images of (**a**) 40 wt.% Pt/S-rGO and (**b**) 80 wt.% Pt/S-rGO.

Figure 6 shows the TEM micrographs and the histograms of Pt NPs for Pt/VC and Pt/S-rGO electrocatalysts. The Pt loading is 20wt%. In the case of Pt/VC, the distribution of Pt NPs on VC is reasonable with slight aggregation and the average particle size of Pt NPs is ~4 nm. For the Pt/S-rGO, the average particle size is ~3.8 nm with symmetrical and uniform distribution. This indicates that S-rGO is an effective supports for Pt NPs. Fig. 7 is the TEM micrographs of 40 wt.% Pt/S-rGO and 80 wt.% Pt/S-rGO electrocatalysts. As can be seen, the average particle size increases slightly with increasing the metal loading. However, the size dispersion is still very uniform even in the case of high Pt loading of 80 wt.%. These results clearly indicate that the sequential functionalization and self-assembly process is very effective for the synthesis of uniform and nano-sized Pt/S-rGO electrocatalysts with high loading owing to the existence of extensive negatively charged sulfonic acid groups on the surface of rGO.

**Figure 8:**
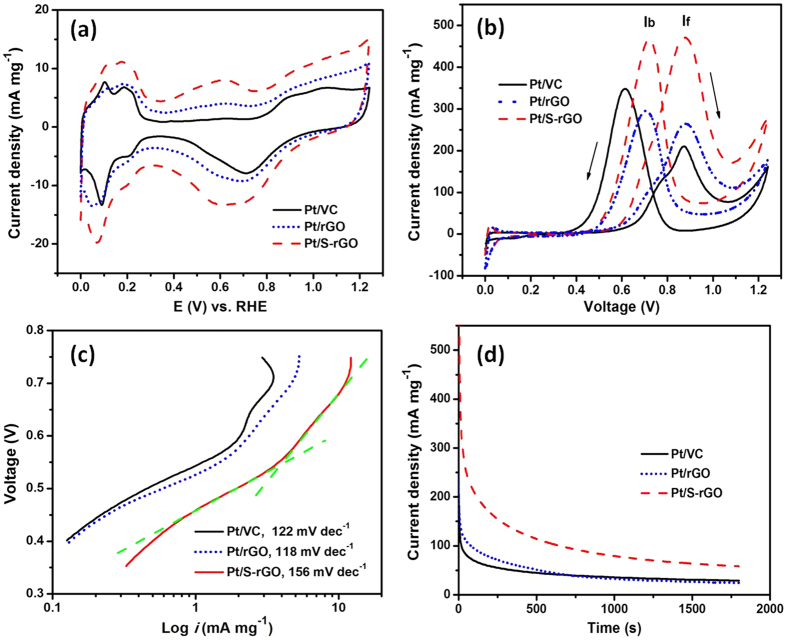
CV curves of the electrocatalysts (**a**) in a N_2_-saturated 0.5 M H_2_SO_4_ solution at a scan rate of 20 mV s^−1^, and (**b**) in a N_2_-saturated 0.5 M H_2_SO_4_ + 1 M CH_3_OH solution at a scan rate of 50 mV s^−1^, (**c**) Tafel plots at a scan rate of 2 mV s^−1^ and (**d**) CA curves at 0.6 V *vs.* SCE for 1800 s in a N_2_-saturated 0.5 M H_2_SO_4_ + 1 M CH_3_OH solution. Pt loading was 20 wt.%. The green dotted lines in (**c**) were used as guide to fit the Tafel plots.

The electrocatalytic activity of the Pt catalysts on the different supports for MOR was examined at 25 °C in a nitrogen-saturated 0.5 M H_2_SO_4_ solution in the absence and presence of 1.0 M CH_3_OH solution and the results are shown in Fig. 8. The electrochemical surface area (ECSA) was obtained from the hydrogen adsorption/desorption area in the CV curves measured in a 0.5 M H_2_SO_4_ solution (Fig. 8a). The higher ECSA in general represents the more active sites for the methanol oxidation. The Pt/S-rGO electrocatalyst shows an ECSA with a value of 394.2 cm^2^ mg^−1^_Pt_, much higher than Pt/VC (286.6 cm^2^ mg^−1^_Pt_) and Pt/rGO (292.4 cm^2^ mg^−1^_Pt_). Considering the similar sizes of the Pt NPs supported on S-rGO and VC supports (see Fig. 6), the significantly high ECSA of the Pt/S-rGO may indicate the synergestic effect between Pt NPs and S-rGO supports. As shown in Fig. 8b, the mass specific peak current density for MOR is 206, 263 and 465 mA mg^−1^ for Pt/VC, Pt/rGO and Pt/S-rGO, respectively. The much higher mass specific catalytic activity of the Pt/S-rGO again indicates that S-rGO plays a significant role to promote the electrocatalytic activity of Pt NPs. Meanwhile, the sulfonic acid groups would be helpful to form hydroxyl groups from water activation, which is essential for removing the metal-poisoning CO molecules from the surfaces of catalysts^37^,^50^. This appears to be supported by the high I_f_/I_b_ (the forward peak current density/the backward peak current density) ratio of Pt/S-rGO (1.0), as compared to I_f_/I_b_ ratio of Pt/VC (0.65) and Pt/rGO (0.89), as the peak current I_f_/I_b_ ratio could be used to estimate the CO tolerance on Pt surface.

The onset potentials are 0.63, 0.60 and 0.57 V for the reaction on Pt/VC, Pt/rGO and Pt/S-rGO, respectively. The negative shift of onset potential indicates an improvement in the electrocatalytic reaction kinetics^51^,^52^. Tafel curves (potential vs. log *i*) were plotted to compare the MOR kinetics of Pt/VC, Pt/rGO and Pt/S-rGO. As shown in Fig. 8c, the Tafel plots can be divided into two nearly linear regions which intersect at approximately 0.55 V for the reaction, indicating a change in the rate determining steps. In the region below 0.55 V, the Tafel slopes of MOR on Pt/VC and Pt/rGO are 122 and 118 mV dec^−1^, very close to the theoretical value predicted for one-electron transfer reaction as rate determining step. However, for Pt/S-rGO, the Tafel slope in the same region is much higher, 156 mV dec^−1^, indicating a faster methanol dehydrogenation even at the relatively low overpotential region^53^.

The stability of the electrocatalysts was evaluated by a chronoamperometry (CA) method at a constant potential 0.6 V vs. SCE (Fig. 8d). As can be seen, polarization current drops significantly with testing time, which is very rapid at the initial stage, followed by a much slower decay. The rapid current decay represents the poisoning effect on the catalyst surface. However, the Pt/S-rGO shows the slowest current decrease among the electrocatalysts studied. After polarized at 0.6 V for 1800 s, the polarization current for the MOR on Pt/S-rGO electrocatalysts is 61 mA mg^−1^_Pt_, significantly higher than 22 mA mg^−1^_Pt_ on Pt/VC and 20 mA mg^−1^_Pt_ on Pt/rGO. This indicates a better tolerance of Pt/S-rGO electrocatalysts to CO or carbonaceous intermediates formed during the MOR.

**Figure 9:**
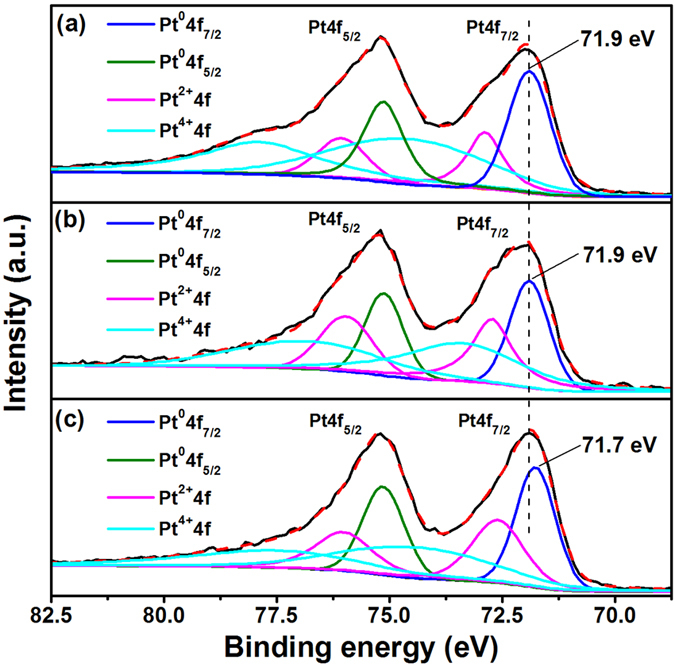
XPS spectra of Pt4f of (**a**) Pt/VC, (**b**) Pt/rGO and (**c**) Pt/S-rGO.

The effect of the grafted sulfonic acid groups on the electronic structure of Pt was further studied by XPS and the results are shown in Fig. 9. The Pt4f spectra of the samples can be fitted with the following components: Pt^0^4f_7/2_, Pt^0^4f_5/2_, Pt^2+^4f_7/2_, Pt^2+^4f_5/2_, Pt^4+^4f_7/2_ and Pt^4+^4f_5/2_. The Pt^0^4f_7/2_ and Pt^0^4f_5/2_ peaks of Pt/S-rGO show a negative shift as compared to that on Pt/VC and Pt/rGO. For example, the binding energy for Pt^0^4f_7/2_ on Pt/S-rGO is 71.7 eV, lower than 71.9 eV observed on Pt/VC and Pt/r-GO. The negative shift of the *d*-band center of Pt is most likely originated to the grafted sulfonic acid group which is electronegative and may donate electrons to Pt. According to the *d*-band center theory, the CO adsorption would be weakened as the *d*-band center decreases^54^. Thus, the negative shift of *d*-band center of Pt NPs on S-rGO presents a weaker oxophilicity, facilitating the removal of the adsorbed poisoning intermediates such as CO_ads_ for MOR^55^. This is supported by the significantly higher I_f_/I_b_ ratio and high current density after polarization at 0.6 V for 1800 s (Fig. 8b, d). The oxidation state of Pt NPs was analyzed and calculated based on the XPS Pt4f spectra, and the results are given in Table 2. The Pt/S-rGO electrocatalyst shows the highest metallic contents of Pt (i.e., Pt(0)) that is important for MOR^56^. The taurine groups assembled on the rGO frameworks significantly enhances the electrocatalytic activity of Pt NPs and facilitate the transfer of electrons and protons for the MOR.

**Figure 10:**
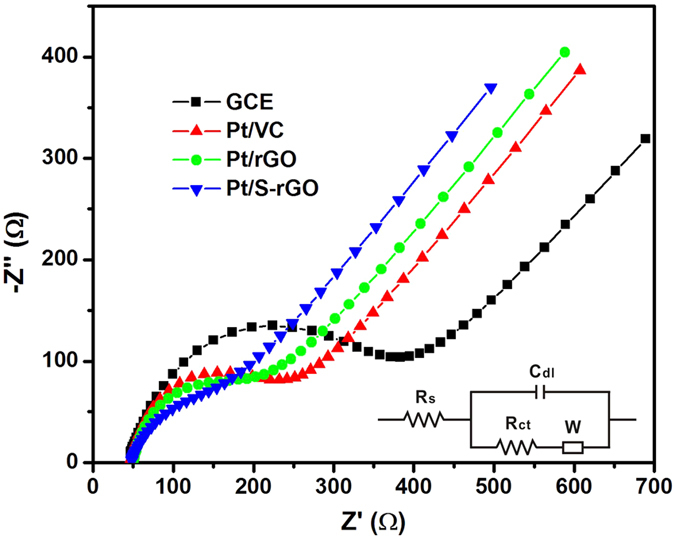
EIS plots of the GCE and different electrocatalysts in 10 mM K_3_[Fe(CN)_6_] + 0.01 M PBS solution. Inset is the equivalent circuit.

The charge transfer capabilities of the different electrocatalysts were further investigated by electrochemical impedance spectroscopy (EIS) in a 10 mM K_3_[Fe(CN)_6_] + 0.01 M PBS solution. In the Nyquist plots as shown in Fig. 10, the semicircular portion at high frequencies corresponds to the charge transfer limited process and the linear portion at low frequency region can be ascribed to the diffusion process. The impedance data were fitted using an equivalent circuit model with NOVA 1.9 software (Metrohm, Switzerland). The electrode charge transfer resistance, *R*_*ct*_ was obtained. The decreased diameter of the semicircle reflects the decrease in the *R*_*ct*_. Based on the equivalent circuit, the *R*_*ct*_ values were determined to be 352, 203, 185 and 158 Ω on the bare GCE, Pt/VC Pt/rGO and Pt/S-rGO electrodes, respectively. These results clearly confirm that *R*_*ct*_ of Pt/S-rGO is significantly lower as compared to that on Pt/VC and Pt/rGO, most likely due to the good ionic conduction ability of sulfonic acid groups grafted on the rGO.

Finally, the synthesis method of the current work was compared to relevant reports on the functionalization of graphene and proposed enhancement mechanism for Pt electrocatalysts (see Table 3). There are similar processes to the current work, but the present study is the first time to report taurine functionalized graphene as Pt catalyst support for MOR and to clarify in details the effects of grafted sulfonic acid groups for the promotion of the dispensability of graphene and catalytic effect on the supported Pt NPs. The existence of sulfonic acid groups on graphene not only enhances the dispensability and stability of graphene, and most important, it modifies the electronic structure and negatively shifts the *d*-band center of Pt, leading to the reduction in charge transfer resistance and enhanced activity of the Pt electrocatalysts for MOR.

## Conclusion

A facile and simple one-pot method was developed to graft taurine molecules onto the basal planes of rGO, and subsequently used as effective Pt catalyst support for MOR via a self-assembly route. The synthesis process was carried out in aqueous solutions without adding any other organic solvents or surfactants. The self-assembled Pt/S-rGO electrocatalyst shows a significantly higher electrocatalytic activity, faster reaction kinetics and a much better stability for MOR in comparison with conventional Pt/VC and Pt/rGO catalysts. The catalytic enhancements could be attributed to the following factors: (1) the presence of large number of sulfuric acid functional group on rGO due to taurine functionalization; (2) uniform deposition of Pt NPs and smaller particle size as result of the self-assembly process; (3) the negative shifted *d*-band center and increased charge transfer ability due to the grafted sulfonic acid groups. The results in this report indicate that it is feasible to functionalize graphene through the newly developed one-pot method and show the promising potential to enhance the practical applications of graphene as effective catalyst support for MOR in DMFCs.

## Methods

### Synthesis of S-rGO

GO was prepared from the natural graphite by a modified Hummers method^57^. The details are provided in the supplementary information. To synthesize S-rGO, 600 mg GO was dispersed into 200 mL deionized (DI) water under ultrasonication. The suspension was centrifuged at 4000 rpm for 30 min to remove the small amount of unexfoliated particles. The obtained supernatant was transferred into a 500 ml flask purged by N_2_, followed by adding 12 g taurine (≥99.5%, Sigma-Aldrich). The mixture was then heated at 60 °C for 36 h under stirring. After cooling to room temperature, a 200 mL with 0.25M sodium borohydride (NaBH_4_, Sigma-Aldrich) aqueous solution was added to the mixture under stirring for 4 h. The mixture was then vacuum filtered through 0.2 μm polycarbonate membrane and washed with DI water to remove the residual ions and excessive raw materials. The as-prepared S-rGO was freeze-dried for 48 h and stored in a dry cabinet for further use.

### Synthesis of Pt/S-rGO electrocatalysts

S-rGO (400 mg) was first sonicated in 200 mL of isopropanol containing 20 mg of PDDA for 2 h and stored at room temperature overnight. Then the as-prepared solution was filtered using 0.2 μm polycarbonate membrane, washed for several times and were dried in a vacuum oven at 70 °C for 10 h. The collected S-rGO-PDDA (50 mg) was mixed with approximate amount of H_2_PtCl_6_ solution under ultrasonication in a beaker, followed by adding 25 mL of 20 mM trisodium citrate under stirring. Then an approximate amount of fresh ice-cold NaBH_4_ solution was dropwise added into the solution under vigorous stirring. After adding the NaBH_4_, large amount of gas bubbles were produced and the color of the solution changed from light yellow to dark brown quickly. The solution was then filtered and washed using polycarbonate membrane. The obtained Pt/S-rGO electrocatalysts were dried in a vacuum oven at 70 °C for 20 h. Pt/S-rGO electrocatalysts with different Pt loading (20, 40 and 80 wt.%) were prepared. The 20 wt.% Pt/VC carbon black were synthesized using the same procedures as described above. Pt NPs supported on rGO were prepared by a chemical reduction method using NaBH_4_ as the reducing agent. Firstly, GO (20 mg) was dispersed in 100 mL DI water by ultrasonication for 30 min before 5 mL H_2_PtCl_6_ solution (10 mg/mL) was added to the solution. Excess NaBH_4_ with a weight of 3 g was slowly added to the solution with magnetic stirring for 24 h at room temperature. The resulting hybrids were separated by filtration and washed with DI water for several times. The Pt loading was controlled at ~20 wt.%.

### Characterization

AFM measurements were performed in the tapping mode using Digital Instruments Nanoscope DI 3100. The morphology of the catalysts was studied by a transmission electron microscope (JEOL 2010) operating at 200 kV. FTIR measurements were carried out with a Perkin Elmer Model GX spectrometer. Raman spectra of the different electrocatalysts were obtained by using a Renishaw^®^ RM1000 Raman spectroscope. XPS measurements were performed on a Kratos Analytical AXIS His spectrometer with a monochromatized Al Ka X-ray source (1486.6 eV photons), in a vacuum of 10^−8^ Torr at a constant dwelling time of 100 ms and pass energy of 40 eV. The anode voltage was 15 mV with a current of 10 mA. All the survey scans and the core-level spectra were obtained at photo-electron take-off angel of 90° (α, with respect to the sample surface). Survey scans were recorded within a range of 0–1200 eV. To compensate for the effects of surface charging, all core-level spectra were referenced to the C1s hydrocarbon peak at 284.5 eV. Elemental analysis was performed using Perkin Elmer Instruments CHNS-O Analyzer. The electrical conductivity of the samples was measured on a resistivity/sheet resistance measurement station (Advanced Instrument Technology, Model: CMT-SR2000N) using a four probe method. The films were prepared by vacuum filtration onto alumina membranes (Millipore, 0.02 μm pore size, 47 mm membrane diameter) supported on a fritted glass holder. After washing with excess DI water, the films were dried in a vacuum oven at 40 °C for 24 h. The thickness of the films was controlled at ~20 μm as measured by using an Alpha-step 500 profilometer. The conductivity was calculated using the formula: *σ* = 1/(*R*_*s*_*t*), where *R*_*s*_ is the resistance (unit: Ω) and *t* is the film thickness (unit: cm). Zeta potential (*ζ*) was measured on a Zetasizer Nano Series 90 at 25 °C, using a DTS 1060 C disposable capillary cell (Malvern Instruments, UK).

### Electrochemical measurements

The electrochemical measurements were carried out in a conventional three-electrode cell using a potentiostat/galvanostat (Autolab PGSTAT30) at ambient temperature. A glassy carbon electrode (GCE, 4 mm in diameter) was used as the working electrode, a platinum wire as the counter electrode, and a saturated calomel electrode (SCE, 0.241 V versus RHE) as the reference electrode. A salt bridge with a Luggin capillary was connected to the reference electrode. To load the electrocatalyst suspension onto GCE, the electrocatalyst powder was ultrasonically mixed in isopropanol to form a homogeneous ink with the catalyst concentration of 5 mg mL^−1^, followed by dropping 10 μL of the electrocatalyst ink onto the surface of GCE. Then, 10 μL 0.5 wt.% Nafion solution was coated onto the surface to fix the electrocatalysts. Without specification, all the electrochemical potentials reported in the present study were given versus RHE. The ECSA of Pt/S-rGO and Pt/VC electrocatalysts was measured in a nitrogen-saturated 0.5 M H_2_SO_4_ solution at a scan rate of 20 mV s^−1^. The electrocatalytic activity for MOR was characterized by the CV measurements in a nitrogen-purged 0.5 M H_2_SO_4_ + 1 M methanol solution at a scan rate of 50 mV s^−1^. The stability was examined by CA tests at a constant potential of 0.6 V vs. SCE. The EIS was conducted in the frequency range of 1 MHz to 1 Hz under the signal amplitude of 10 mV in 10 mM K_3_[Fe(CN)_6_] + 0.01 M phosphate (KH_2_PO_4_) buffered saline solution (PBS, pH = 7.4).

## Additional Information

**How to cite this article**: Lu, J. *et al*. Self-assembled platinum nanopartilces on sulfonic acid-grafted graphene as effective electrocatalysts for methanol oxidation in direct methanol fuel cells. *Sci. Rep.* **6**, 21530; doi: 10.1038/srep21530 (2016).

## Supplementary Material

Supplementary Information

## Figures and Tables

**Table 1:** Elemental analysis, electrical conductivity and zeta potential of VC, GO, rGO and S-rGO.

Sample	C (wt.%)	O (wt.%)	H (wt.%)	N (wt.%)	S (wt.%)	*σ* (S·cm^−1^)	*ζ*(mV)
VC	98.50	1.24	<0.10	<0.10	<0.10	2.1	−24.8
GO	52.65	43.17	4.08	<0.10	<0.10	2.4 × 10^−4^	−27.6
rGO	85.74	13.06	1.20	<0.10	<0.10	5.2	−10.4
S-rGO	74.63	15.42	2.52	2.26	5.17	4.3	−64.2

**Table 2:** Summary of oxidation states for Pt species of different electrocatalysts obtained from XPS analysis.

Sample	Pt species on surface	Binding energy of Pt4f_7/2_(eV)	Relative intensity (%)
Pt/VC	Pt(0)	71.9	33.36
	Pt(II)	72.9	18.31
	Pt(IV)	74.6	48.33
Pt/rGO	Pt(0)	71.9	31.62
	Pt(II)	72.7	28.06
	Pt(IV)	73.5	40.32
Pt/S-rGO	Pt(0)	71.7	38.45
	Pt(II)	72.6	27.16
	Pt(IV)	74.3	34.39

**Table 3:** Comparison of current work and relevant reports on the functionalization of graphene and proposed enhancement mechanism.

Ref.	Species and method for functionalizing graphene	Synthesis method for catalysts	Mechanism for the enhanced performance
27	—	Pulsed galvanostatic electrodeposition	Synergistic effects
28	—	Magnetron sputtering	Porous honeycomb-like structure; Large surface area; Enhanced interactions
29	—	Chemical reduction	Bifunctional effect between Pt NPs and remaining oxygenated groups on graphene
30	Nitrogen-doped; Chemical reduction	Chemical reduction	Smaller particle size; Modulated electronic properties of Pt
31	Boron-doped; Thermal annealing	Microwave-assisted polyol process	Uniform deposition of Pt NPs; Lower d-band center
32	Nitrogen-doped; Hydrothermal	Hydrothermal	Smaller particle size; Stronger binding energy between Pt and N-doped graphene
33	Nitrogen-doped and ZrO_2_-decorated; Atomic layer deposition	Chemical reduction	Unique structure
34	Nitrogen-doped; Ultrosonic cavitation assisted hydrogen implosion	Chemical reduction	Enhanced electronic conductivity; Special structure
Current work	Grafted with taurine molecules; One-pot method	Self-assembly	Smaller particle size and uniform distribution; Lower *d*-band center; Enhanced charge transfer ability
