# Supplementary material for: Self-assembled platinum nanoparticles on sulfonic acid-grafted graphene as effective electrocatalysts for methanol oxidation in direct methanol fuel cells
Source: Sci Rep. 2016 Feb 15;6:21530. doi: 10.1038/srep21530 (PMC4753497; doi:10.1038/srep21530)
Supplement: Supplementary Information [file srep21530-s1.doc]

**Supplementary Information**

**Self-assembled platinum nanoparticles on sulfonic acid-grafted graphene as effective electrocatalysts for methanol oxidation in direct methanol fuel cells**

Jinlin Lu^1,*^, Yanhong Li^1^, Shengli Li, San Ping Jiang^2,*^

^1^School of Materials and Metallurgy, University of Science and Technology Liaoning, Anshan 114051, P. R. China.

^2^Fuels and Energy Technology Institute & Department of Chemical Engineering, Curtin University, Perth, WA 6102, Australia

**Synthesis of GO and rGO**

2.5 g Graphite and 2.5 g NaNO_3_ were mixed with 120 ml H_2_SO_4_ in a 500 mL flask. The mixture was stirred for 30 min in an ice bath and 7.5 g of KMnO_4_ was slowly added to the suspension under vigorous stirring. The ice bath was removed and the mixture was then stirred at 35°C for 24 h. Afterwards, 150 ml of DI H_2_O was slowly added to the pasty mixture still under vigorous stirring. The reaction temperature was observed to rapidly increase to over 90 °C with effervescence. After 30 min, another 500 ml DI water was added, and then 1.5 mL of 30 wt.% H_2_O_2_ aqueous solution. For preliminary purification, the mixture was first washed with 5 wt.% HCl aqueous solution, followed by DI water for five times to remove residual acid and salts. The yellow mixture is centrifuged 30 min at 11,000 rpm. The obtained GO was freeze-dried for 48 h and stored in a dry cabinet for further use. The rGO was also prepared by reducing the as-prepared GO using a NaBH_4_ aqueous solution at room temperature.


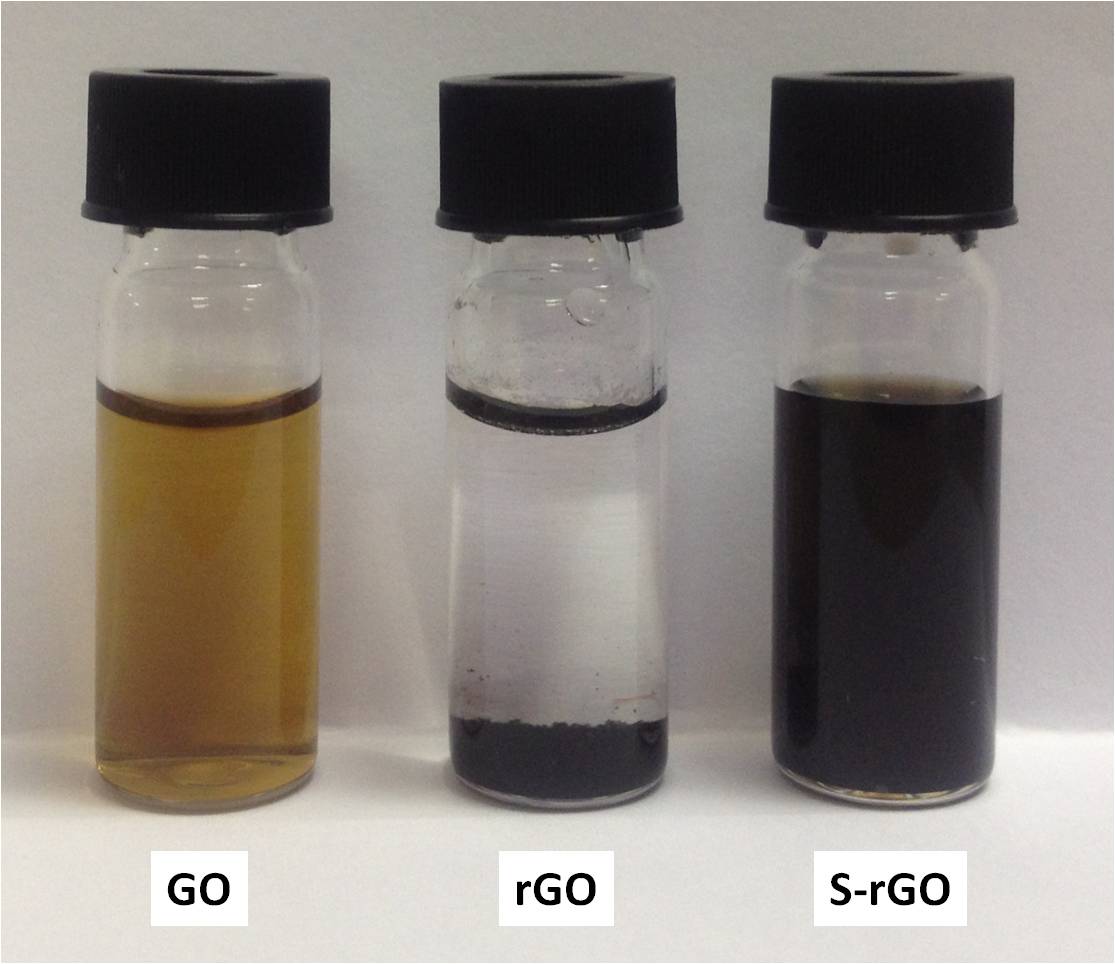


Figure S1∣Optical photographs of GO, rGO and S-rGO dispersed in water after storing for 3 months.


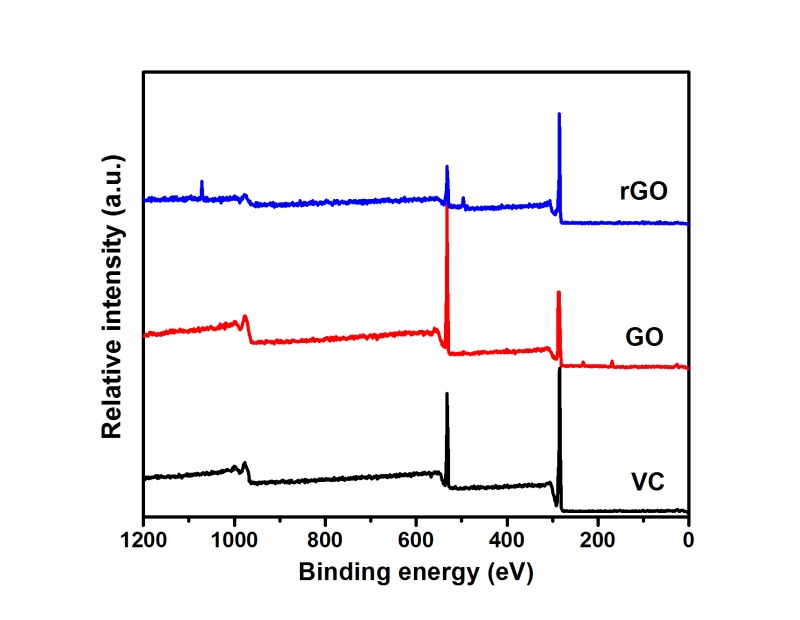


Figure S2∣XPS survey scan of VC, GO and rGO.


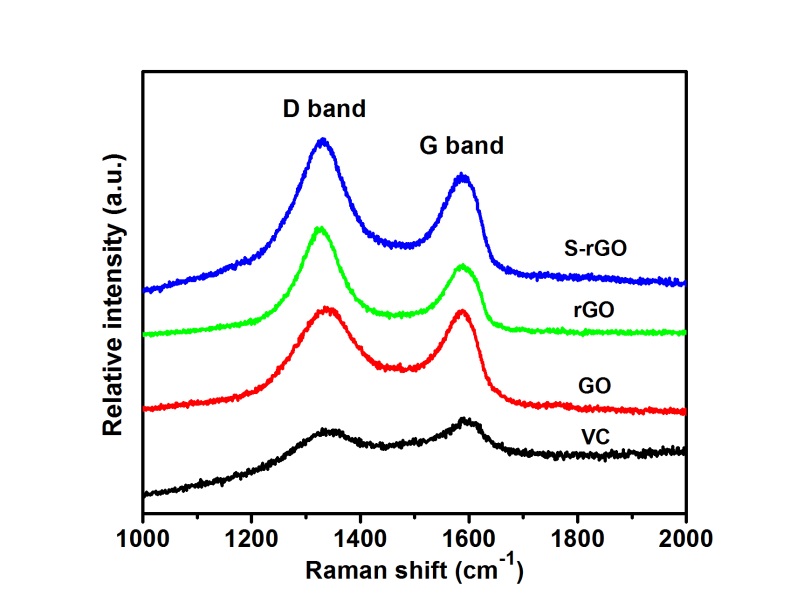


Figure S3∣Raman spectra of VC, GO, rGO and S-rGO.


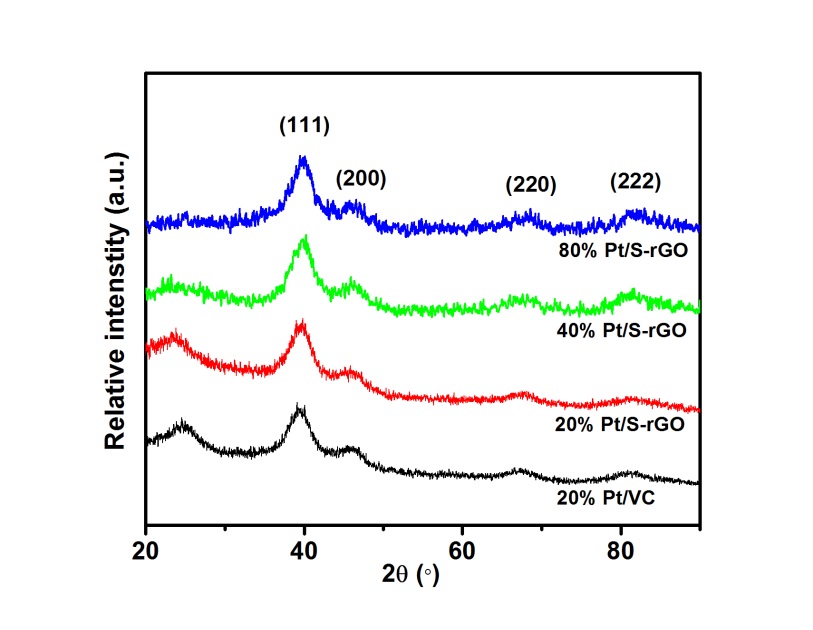


Figure S4∣XRD patterns of the different electrocatalysts.

X-R diffraction (XRD) patterns of the catalysts were obtained on an XRD-6000 diffractometer employing Cu K_α_ radiation (λ=0.15418 nm) at a scan rate of 5° min^-1^ and the scan range from 20° to 80°.

The XRD patterns (Fig. S4) are used to determine the presence of crystalline Pt and the average crystallite sizes on the supports. The peaks around 39.7° (111), 46.1° (200) and 67.8° (220) indicate that Pt is loaded on to the supports. The average crystalline size (*D*) can be calculated from the Scherrer formula: *D*=0.9·*λ*/(*β*·cos*θ*), where *λ* is X-ray wavelength (λ=0.15418 nm), *β* is the width of the diffraction peak at half height in radians, and *θ* is the angle at the position of the peak maximum. The as-obtained results are quite similar to the data measured by TEM.


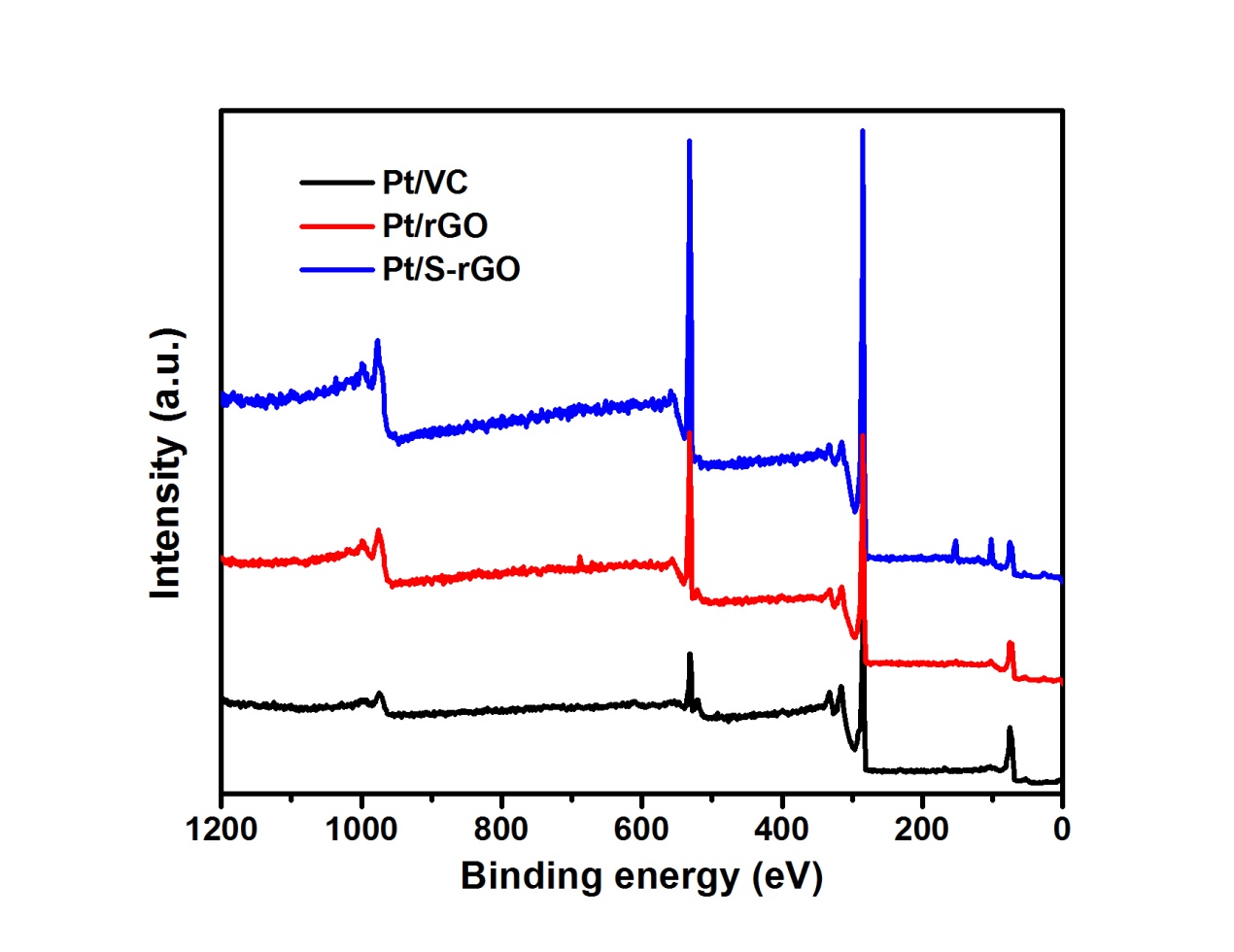


Figure S5∣XPS survey scan of Pt/VC, Pt/rGO and Pt/S-rGO.
